# Distal Radial Fractures with Scaphoid Fractures

**DOI:** 10.1055/s-0044-1785464

**Published:** 2024-04-10

**Authors:** Caio Kzan Geyer Nogueira, Vinícius Ynoe de Moraes, Lucas Pereira Sarmento, Luís Renato Nakachima, João Baptista Gomes dos Santos, João Carlos Belloti

**Affiliations:** 1 Disciplina de Cirurgia da Mão e Membro Superior, Escola Paulista de Medicina, Universidade Federal de São Paulo, São Paulo, SP, Brasil; 2 Ortopedia e Traumatologia, Escola Paulista de Medicina, Universidade Federal de São Paulo, São Paulo, SP, Brasil

**Keywords:** general surgery, functional status, radius fractures, wrist fractures, scaphoid bone, therapeutics

## Abstract

**Objective** This study evaluated the epidemiological data and functional outcomes from patients with concomitant distal radial and scaphoid fractures treated in a single center specialized in hand surgery. Functional outcomes analysis used validated instruments.

**Methods** Patients diagnosed with distal radial and scaphoid fractures treated from January 2011 to December 2021 underwent assessments using the Disabilities of the Arm, Shoulder and Hand (DASH), Patient-Rated Wrist Evaluation (PRWE), Visual Analog Scale (VAS) for pain, goniometry, radiographic consolidation, and complications six months after surgery.

**Results** The study included 23 patients, 73.9% men and 26.1% women. Most (56.5%) fractures occurred on the right side, and 43.5% happened on the left side. Treatment of most (56%) distal radial fractures used a locked volar plate. Functional assessment by PRWE resulted in a mean score of 35.9 points (range, 14 to 71 points), while DASH showed a mean score of 37.8 points (range, 12 to 78 points). The mean VAS was 2.33 during activities (range, 0.6 to 6.2).

**Conclusion** Distal radial fractures associated with scaphoid fractures resulted from high-energy trauma, and most patients were males. There was a low rate of complications with surgical treatment, and the patients had satisfactory functional evolution with a low level of pain.

## Introduction

Isolated distal radial fractures are common, and their incidence in the population is high. 1 Meanwhile, scaphoid fractures are the most frequent among carpal bones. However, the simultaneous occurrence of distal radial and scaphoid fractures is rare 2 , and it often results from high-energy trauma. The literature reports its incidence in 0.5 to 5% of distal radial fractures. 3

Concomitant radial and scaphoid fractures represent a treatment challenge. At surgery, scaphoid fractures require anatomical reduction and compression fixation. On the other hand, distal radial fractures require traction for proper reduction and fixation. 4 5 This implies careful operative planning adapted to each fracture type. Postoperative management also involves conflicting principles: scaphoid fractures require a longer immobilization period for consolidation, but distal radial fractures require a shorter immobilization period followed by early rehabilitation to avoid wrist joint stiffness. 6

In 2022, in a systematic review of 20 case series about concomitant distal radial da scaphoid fractures, Blackburn et al. 3 noted that the main limitation of these studies was the lack of functional and quality of life assessments using validated instruments, such as the Disabilities of the Arm, Shoulder, and Hand (DASH) questionnaire and the Patient-Rated Wrist Evaluation (PRWE) questionnaire.

The primary objective of this study was to evaluate the functional outcomes of patients with concomitant distal radial and scaphoid fractures. The secondary objective was to analyze the epidemiological aspects of patients treated in a single center specialized in hand surgery, using validated instruments to determine their functional outcomes.

## Methods

We collected medical records from January 2011 to December 2021 of all patients diagnosed with an acute distal radial fracture (up to 15 days) registered in the surgical schedule of the only medical center specialized in hand surgery. Of the patients initially selected (N = 957), only those diagnosed with a concomitant distal radial and ipsilateral scaphoid fracture (N = 38) who underwent surgical treatment were included.

Patients with fractures in other carpal bones, complete injuries of the scapholunate ligament (visualized on stress radiographs with an opening greater than 5 mm), fractures with neurotendinous injuries, fractures with loss of skin coverage, and patients with sequelae of previous traumatic, degenerative, or both injuries with functional deficits in the affected hand or contralateral wrist were excluded from this study.

The same surgeon specialized in hand surgery and microsurgery operated on all selected patients and followed the same fracture fixation steps, first performing scaphoid fixation using a percutaneous method and then a radial fracture fixation. The surgeon followed these steps regardless of the method chosen.

After identifying the patients, we recruited those selected by telephone, telegram, or both for an in-person clinical assessment of the outcomes analyzed in this study.

The clinical outcomes evaluated, i.e., PRWE and DASH questionnaires, were the primary outcomes for functional assessment. Secondary outcomes included active and passive wrist range of motion measurements (flexion, extension, radial deviation, ulnar deviation, pronation, supination), grip strength measured with a Jamar dynamometer, pain measurement using the Visual Analog Scale (VAS), and radiographic evaluation. All patients were assessed for a minimum follow-up period of 6 months.

The DASH questionnaire is a tool to assess the impact of the disease on the function of the affected upper limb. It has 37 questions about upper limb function, and its score ranges from 0 (no complaints) to 100 (highly limiting conditions for the limb). 7

The PRWE questionnaire consists of 15 questions specifically about wrist function. It has five questions about pain, six about function in specific activities, and four about daily activities. The final score ranges from 0 (no complaints) to 10 (highly limiting conditions for the limb). 8

The radiographic analysis included the evaluation of orthogonal radiographs of the wrist and measured the radiographic values of radial height, ulnar variance, radial angulation, and volar inclination. Fracture consolidation and the occurrence of osteoarthritis were also determined according to the Knirk and Jupiter classification, 9 in which grade 0 represents the absence of osteoarthritis signs, grade I indicates decreased joint space, grade II implies a higher decrease in joint space with osteophyte formation, and grade III denotes direct bone contact with the osteophyte and subchondral cyst formation. The researcher in charge of data analysis conducted the radiographic evaluation.

Complications include any complications during the intervention or clinical follow-up of patients requiring surgical treatment and not foreseen in the initial surgery. The definition of pseudarthrosis was the absence of signs of clinical and radiographic consolidation of the distal radial or scaphoid fracture after 6 months of osteosynthesis 10 at radiographs in orthogonal planes.

The Ethics Committee approved this project under CAAE number 60074522.2.0000.5505.

For statistical analysis, the data were stored in an Excel® for Mac spreadsheet and subsequently imported into the SPSS® 23 for Mac Software. Descriptive statistics of categorical data used absolute frequency of occurrence and its respective proportion. Continuous data were analyzed for distribution subjectively using a histogram and objectively using the Shapiro-Wilk test. Due to the non-symmetric nature of continuous data, the description employed median and the 25th and 75th percentile values.

The non-parametric Spearman correlation test determined correlations between variables. For statistical inference, p-values <0.05 were considered for type I error. The Cohen index 11 assessed the magnitude of the correlations using the following classification: values >0.8 indicate great magnitude, values ranging from 0.5 to 0.8 indicate medium magnitude, and values ranging from 0.2 to 0.3 show small magnitude.

## Results

**Fig. 1:**
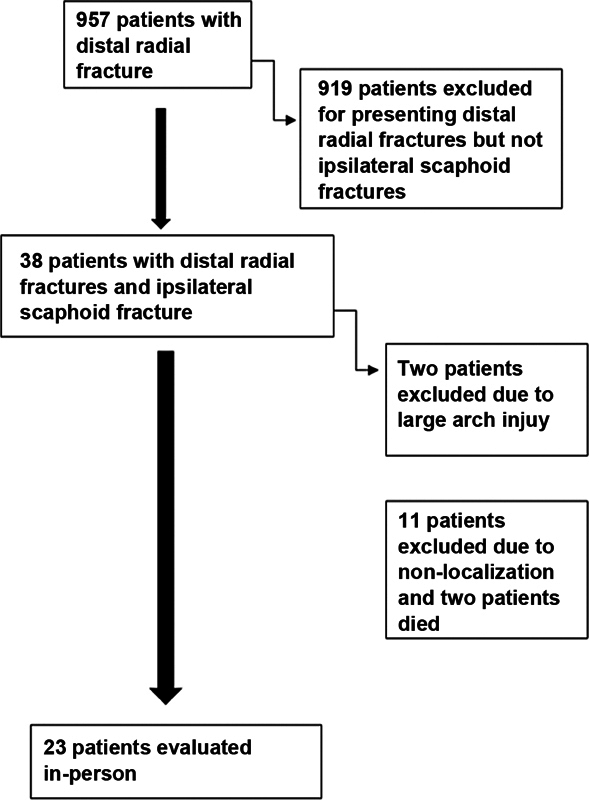
Organizational chart of patient acquisition.

We analyzed 957 medical records of patients undergoing surgical treatment from January 2011 to December 2021 due to a distal radial fracture. Of these patients, 38 also presented an ipsilateral scaphoid fracture, resulting in a 3.7% prevalence. We excluded two records because their fractures were at the great arch, and the final sample had 36 patients. After telephone and telegram attempts, we could not locate 11 patients, and two had died from unrelated causes. In the end, 23 patients were evaluated ( Fig. 1 ).

Concerning demographic characteristics, there were 73.9% men and 26.1% women, with 82.6% right-handed and 7.4% left-handed subjects. Regarding the lesion side, the distribution was similar, with 56.5% fractures on the right side and 43.5% on the left side. Treatment of most distal radial fractures used a volar locked plate (56%), followed by a dorsal locked plate (21.7%), while the remaining patients received other fixation methods. Scaphoid fracture fixation mostly employed compressive screws, and only two patients underwent fixation with Kirschner wires ( Table 1 ).

**Table 1:** General characteristics and treatment from 23 patients with distal radial and scaphoid fractures

	n	%
Gender		
Female	6	26.1
Male	17	73.9
Total	23	100.0
Dominance		
Right	19	82.6
Left	4	17.4
Total	23	100.0
Lesion side		
Right	13	56.5
Left	10	43.5
Total	23	100.0
Radius fixation method		
External fixation	2	8.7
Kirschner wire	1	4.34
Hebert	2	8.7
Dorsal plate	5	21.7
Volar plate	13	56.5
Total	23	100.0
Scaphoid fixation method		
Kirschner wire	2	8.7
Anterograde screw	10	43.5
Retrograde screw	11	47.8
Total	23	100.0

Regarding the results of the questionnaires, the mean PRWE score was 35.9 points (range, 14 to 71), the mean DASH score was 37.8 points (range, 12 to 78), and the mean VAS was 2.33 during activities (range, 0.6 to 6.2).

**Fig. 2:**
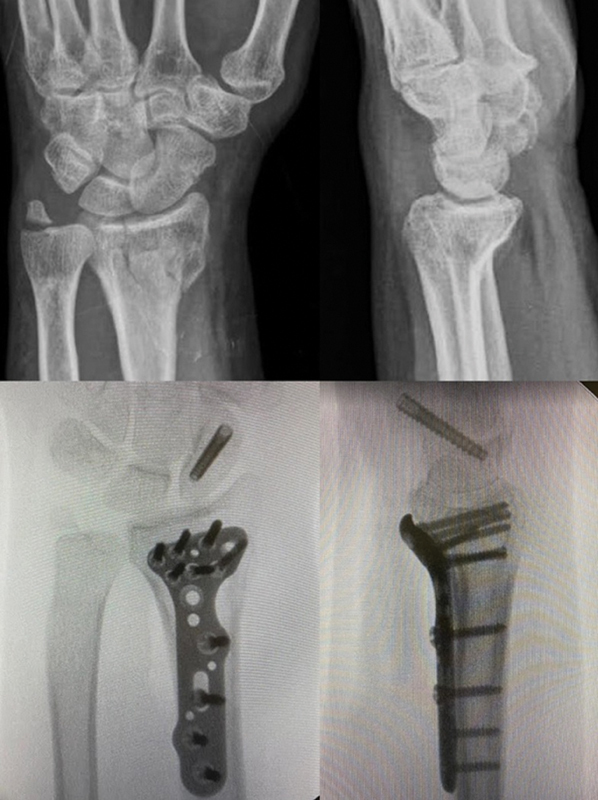
45-year-old male patient with a history of high-energy trauma. Treatment consisted of osteosynthesis of the distal radius with a volar plate and osteosynthesis of the scaphoid with a compressive screw.

Analysis of radiographic aspects revealed an average of 0.65 mm (−2 mm to 3 mm) for ulnar variance, 11.43 mm (14 mm to 8 mm) for radial height, 20.57 degrees (24 degrees to 18 degrees) for radial tilt, and 15.30 degrees (24 degrees to −6 degrees) for volar tilt. The AO/ASIF system for distal radial fractures classified two fractures as A3, 11 as C2, and ten as C3. Per the IDEAL classification system, 12 20 patients were potentially unstable, and three were complex. When evaluating the presence of arthrosis in the participants' wrists using the Knirk-Jupiter classification, twelve subjects were grade 0, nine were grade 1, and only two were grade 2 ( Fig. 2 and Table 2 ).

**Table 2:** Functional assessment, classification, and pain in 23 patients with distal radial and scaphoid fractures

	Mean	Standard deviation	Median	1 ^st^ interquartile range	3 ^rd^ interquartile range
Immobilization time (weeks)	8.00	2.54	8.00	6.00	8.00
Time to resume work (weeks)	18.81	20.52	12.00	12.00	16.00
Active flexion (degrees)	50.09	10.37	52.00	42.00	58.00
Passive flexion (degrees)	54.78	9.89	56.00	48.00	63.00
Active extension (degrees)	54.70	11.59	54.00	49.00	62.00
Passive extension (degrees)	59.65	11.87	58.00	53.00	69.00
Active radial deviation (degrees)	9.96	2.93	10.00	8.00	10.00
Passive radial deviation (degrees)	12.52	2.91	12.00	12.00	14.00
Active ulnar deviation (degrees)	19.04	5.08	18.00	16.00	20.00
Passive ulnar deviation (degrees)	22.70	5.10	22.00	20.00	24.00
VAS in rest (centimeters)	0.82	1.02	0.30	0.05	1.25
VAS during activity (centimeters)	2.33	1.71	1.40	1.20	3.20
PRWE	35.96	14.03	34.00	27.00	41.50
DASH	37.83	14.77	34.00	30.00	40.00
Ulnar variance (millimeters)	0.65	1.56	1.00	−1.00	2.00
Radial height (millimeters)	11.43	1.80	12.00	10.00	12.50
Radial tilt(degrees)	20.57	1.97	20.00	19.50	22.00
Volar tilt (degrees)	15.30	6.89	16.00	14.50	19.00
Jamar on the operated side (N/Kg)	27.32	7.29	30.34	22.67	32.67	
Jamar on the non-operated side (N/Kg)	31.46	9.92	33.33	24.67	36.67	

VAS, Visual analog scale; DASH, Disabilities of the Arm, Shoulder and Hand; PRWE, Patient-Rated Wrist Evaluation.

Of the 23 participants, only three had complications. The first was a surgical wound infection one week post-operatively, treated with antibiotics and evolving to complete resolution. Two patients required removal of the radius synthesis due to tenosynovitis of the flexor tendons associated with the blocked volar plate.

The mean immobilization time was 8 weeks (range, 6 to 12 weeks), while the average return to work took 18.81 weeks (range, 8 weeks to 1 year). One patient required 2 years and 3 months before returning to work due to a complex regional pain syndrome requiring treatment in conjunction with the specialized pain team ( Table 3 ).

**Table 3:** Arthrosis presence per the Knirk-Jupiter classification

Knirk-Jupiter classification		
G0	9	39.1%
G1	9	39.1%
G2	5	21.7%
G3	0	0.0%

According to the Spearman correlation index, there was a pattern of great magnitude in the measured range of motion values (active flexion, active extension, active radial deviation, active ulnar deviation) ( Table 4 ). The correlation index between PRWE and DASH questionnaires was 0.773, considered as medium magnitude. The same occurred between PRWE and VAS during activities. The other correlations exhibited effects of small magnitude effects ( Table 5 ).

**Table 4:** Correlations with motion arc measurements

		Passive flexion	Passive extension	Passive radial deviation	Ulnar radial deviation
Active flexion	Correlation coefficient	0.990 *	–	–	–
	p-value	<0.01			
Active extension	Correlation coefficient	–	0.978 *	–	–
	p-value		<0.01		
Active radial deviation	Correlation coefficient	–	–	0.845 *	–
	p-value			<0.01	
Ulnar active deviation	Correlation coefficient	–	–	–	0.826 *
	p-value				<0.01

* Valores acima de 0.8 são considerados valores de grande magnitude.

**Table 5:** Correlações dos resultados dos questionários PRWE, DASH e EVA

		PRWE	DASH	EVA Repouso	Eva Esforço
PRWE	Correlation coefficient	–	0.773 *	0.462	0.739 *
	p-value		<0.01	0.026	<0.01
DASH	Correlation coefficient	–	–	0.210 **	0.418 **
	p-value			0.336	0.047
VAS in rest	Correlation coefficient	–	–	–	0.642 *
	p-value				<0.01
VAS during activity	Correlation coefficient	0.739 *	0.418 **	0.642 *	–
	p-value	<0.01	0.047	<0.01	

VAS, Visual analog scale; DASH, Disabilities of the Arm, Shoulder and Hand; PRWE, Patient-Rated Wrist Evaluation.

* Medium magnitude values.

** Small magnitude values.

The correlation had a great magnitude in the measured range of motion values (active flexion, active extension, active radial deviation, active ulnar deviation). The correlation index of PRWE and DASH questionnaires had a medium magnitude, of 0.773, just like the correlation between PRWE and VAS under activity. The other correlations showed small effect magnitudes. Table 4 and Table 5 show these correlations.

## Discussion

Among distal radial fractures, the concomitant association with ipsilateral scaphoid fractures is rare, with an incidence ranging from 0.5% to 5% according to the literature. All patients in this series suffered high-energy trauma mechanisms. Most subjects were males (73.4%), in the fourth decade of life (average age, 38.4 years old), consistent with the literature. 2 4 7 9 13 14 15 16 17 18 19 The fractures presented a comminuted pattern, strengthening the hypothesis of high-energy trauma, corresponding to subgroup C per the AO/ASIF Classification (21 patients). Most (86%) scaphoid fractures presented a simple line at waist level without displacement, and the remaining 14% of fractures occurred at the proximal pole of the scaphoid.

In the present study, including 10 years (2011-2021) of records, we evaluated 957 patients with surgical distal radial fracture, including 36 subjects presenting a concomitant, ipsilateral scaphoid fracture, resulting in an incidence of 3.7%. Among these, we included 23 patients in this study to analyze their outcomes. This represents a significant sample given the low prevalence of this injury and the researched literature, which reportedly has only five publications with considerable sample sizes. 2 3 4 13 19 The largest series identified was published by Vukov et al. (1988) 13 and had 26 patients. However, all subjects received conservative treatment, which resulted in a high rate of complications (57%).

In this study, a single surgeon performed the surgical treatment of the injuries; therefore, the surgical tactic for lesion treatment always started with the distal radial fracture followed by scaphoid fixation using a percutaneous, antegrade, or retrograde technique chosen for each case. We adopted this strategy since most scaphoid fractures were non-displaced, not requiring reduction maneuvers for fixation.

Our results demonstrated that all patients presented consolidation of both radius and scaphoid fractures. Only one patient faced difficulties in returning to his original work activity due to complex regional pain syndrome. Similarly, Fowler et al. (2018), 4 in a series of 23 patients treated surgically, obtained a scaphoid consolidation rate of 93%. A single subject had a displaced scaphoid fracture and, at the postoperative evaluation, only one case did not present consolidation (corresponding to a patient with an ipsilateral brachial plexus injury).

The complication rate presented in this study was 13%, reinforcing the effectiveness of surgical treatment for patients' functional outcomes. Blackburn et al. (2022) 3 in a systematic review of the literature, identified 20 case series involving concomitant ipsilateral fractures of the scaphoid and distal radius. This review noted the association with high-energy mechanisms and supported the need for a surgical approach for this injury. However, the authors also realized the scarcity of studies presenting adequate parameters for comparison with postoperative assessment, identifying a single paper using questionnaires such as PRWE.

Given the low prevalence of this injury, prospective randomized multicenter studies are needed to provide more robust evidence. Although our study was retrospective, we contributed to the analysis of outcomes by employing validated functional assessment tools and demonstrating that both DASH and PWRE were consistent in their evaluations, an association previously not identified in the literature review previously mentioned. These instruments allowed us to statistically identify a correlation index of 0.773, indicating that both instruments should be considered when evaluating these patients in future studies.

## Conclusion

We found that distal radial fractures associated with scaphoid fractures were more prevalent in young male patients and resulted from high-energy trauma. Surgical treatment proved effective according to the evaluation of clinical, radiographic, and functional outcomes and had a low rate of complications.
