# Supplementary material for: Distal Radial Fractures with Scaphoid Fractures
Source: Rev Bras Ortop (Sao Paulo). 2024 Apr 10;59(2):e247–53. doi: 10.1055/s-0044-1785464 (PMC11006516; doi:10.1055/s-0044-1785464)
Supplement: Related texts

# Research article: Fraturas do rádio distal associadas à fratura do escafoide

**Keywords:** cirurgia geral, estado funcional, fraturas do rádio, fraturas do punho, osso escafoide, terapêutica

## Abstract

**Objetivo** Avaliar os resultados epidemiológicos e funcionais dos pacientes que apresentaram fraturas concomitantes do rádio distal e do escafoide e foram tratados em um único centro especializado em cirurgia da mão, através de instrumentos validados para analisar os desfechos funcionais desses pacientes.

**Métodos** Foram avaliados os pacientes com diagnóstico de fratura do rádio distal e escafoide tratados de janeiro de 2011 até dezembro de 2021, através dos questionários *Disabilities of the Arm, Shoulder and Hand* (DASH), *Patient Rated Wrist Evaluation* (PRWE) e Escala Visual Analógica da dor (EVA); goniometria; consolidação radiográfica; complicações em seis meses de pós-operatório.

**Resultados** Vinte e três pacientes foram incluídos no estudo, sendo 73,9% homens e 26,1% mulheres; 56,5% das fraturas ocorreram à direita e 43,5% à esquerda. A maioria das fraturas do rádio distal foi tratada com placa volar bloqueada, totalizando 56%. Na avaliação funcional pelo PRWE, obteve-se média de 35,9 pontos (variação de 14 a 71 pontos) e pelo DASH média de 37,8 pontos (variação de 12 a 78 pontos). A EVA apresentou uma média de 2,33 durante a atividade (variação de 0,6 a 6,2).

**Conclusão** Verificou-se que as fraturas do rádio distal associadas a fraturas do escafoide foram causadas por traumas de alta energia, com o sexo masculino mais acometido. Houve baixo índice de complicações com tratamento cirúrgico e os pacientes tiveram evolução funcional satisfatória, com baixo índice de dor.

## Introdução

As fraturas isoladas do rádio distal são comuns, apresentando alta incidência na população, 1 enquanto as fraturas do escafoide são as mais frequentes entre os ossos do carpo. No entanto, a ocorrência simultânea de fraturas da extremidade distal do rádio e do escafoide é uma condição rara, geralmente decorrente de traumas de alta energia, 2 com uma incidência relatada na literatura de 0,5 a 5% das fraturas do rádio distal. 3

Fraturas concomitantes do rádio e do escafoide representam um desafio no tratamento. Durante a intervenção cirúrgica, as fraturas do escafoide demandam uma redução anatômica e fixação com compressão. Por outro lado, as fraturas do rádio distal requerem tração para alcançar a adequada redução e fixação. 4 5 Isso implica um planejamento operatório cuidadoso, adaptado a cada tipo de fratura. O manejo pós-operatório também envolve princípios conflitantes: as fraturas do escafoide exigem um período mais prolongado de imobilização para a consolidação, enquanto as do rádio distal necessitam de menor período de imobilização seguido de reabilitação precoce, a fim de evitar a rigidez articular do punho. 6

Em 2022, Blackburn et al. 3 realizaram uma revisão sistemática abrangendo 20 séries de casos relacionados a fraturas concomitantes da extremidade distal do rádio e do escafoide. Observou-se que a principal limitação desses estudos foi a falta de avaliações funcionais e de qualidade de vida por meio de instrumentos validados, como o questionário *Disabilities of the Arm, Shoulder and Hand* (DASH) e o questionário *Patient Rated Wrist Evaluation* (PRWE).

O objetivo deste estudo é avaliar primariamente os resultados funcionais dos pacientes que apresentaram fraturas concomitantes do rádio distal e do escafoide e secundariamente os aspectos epidemiológicos de pacientes tratados em um único centro especializado em cirurgia da mão, através de instrumentos validados para analisar os desfechos funcionais desses pacientes.

## Métodos

Foram coletados prontuários no período de janeiro de 2011 a dezembro de 2021 de todos os pacientes com diagnóstico de fratura aguda do rádio distal (até 15 dias), registrados na agenda cirúrgica do único centro médico especializado em cirurgia da mão. Dos pacientes inicialmente selecionados (N = 957), foram incluídos somente aqueles com diagnóstico de fratura concomitante do rádio distal e do escafoide ipsilateral (N = 38), que foram submetidos a tratamento cirúrgico.

Pacientes com fraturas associadas a outros ossos do carpo, lesões completas do ligamento escafo-semilunar (visualizadas nas radiografias em estresse com abertura superior a 5mm), fraturas com lesões neurotendíneas associadas, fraturas com perda de cobertura cutânea, bem como pacientes com sequelas de lesões traumáticas e/ou degenerativas prévias com déficits funcionais na mão acometida ou no punho contralateral foram excluídos deste estudo.

Todos os pacientes selecionados foram operados pelo mesmo cirurgião especialista em mão e microcirurgia, que seguiu os mesmos passos de fixação das fraturas, realizando primeiro a fixação do escafóide por método percutâneo seguido da fixação da fratura do rádio, mantendo estes passos independente do método escolhido.

Após a identificação dos pacientes, os selecionados foram recrutados por contato telefônico e/ou telegrama para avaliação clínica presencial dos desfechos analisados neste estudo.

Quanto aos desfechos clínicos avaliados **,** adotaram-se os questionários PRWE e DASH como desfechos primários para avaliação funcional. Os desfechos secundários incluíram medidas de amplitude de movimento do punho ativas e passivas (flexão, extensão, desvio radial, desvio ulnar, pronação, supinação), força de preensão medida por dinamômetro de Jamar, mensuração da dor por meio da Escala Visual Analógica (EVA) e avaliação radiográfica. Todos os pacientes foram avaliados com um seguimento mínimo de seis meses.

O questionário DASH é uma ferramenta usada para avaliar o impacto da doença na função do membro superior acometido. Composto por 37 perguntas pertinentes para a função do membro superior, o escore varia de 0 (sem queixas) a 100 (condições altamente limitantes para o membro). 7

O questionário PRWE consiste em 15 perguntas pertinentes, sendo específico para a função do punho e dividido em cinco perguntas sobre dor; seis perguntas sobre função em atividades específicas e quatro perguntas sobre atividades diárias. A pontuação final varia de 0 (sem queixas) a 10 (condições altamente limitantes para o membro). 8

A análise radiográfica compreendeu a avaliação de radiografias ortogonais do punho, com mensuração dos valores radiográficos da altura radial, variância ulnar, angulação radial e inclinação volar. Também foi determinada a consolidação da fratura e a ocorrência de artrose, conforme a classificação de Knirk e Jupiter, 9 na qual o grau 0 representa ausência de sinais de artrose, grau I indica diminuição do espaço articular, grau II implica franca diminuição do espaço articular com formação de osteófitos, e grau III denota contato ósseo direto com formação de osteófitos e cistos subcondrais. A avaliação radiográfica foi conduzida pelo pesquisador responsável pela análise dos dados.

Foram definidas como complicações quaisquer intercorrências durante a intervenção ou o seguimento clínico dos pacientes que necessitaram de tratamento cirúrgico não previsto no tratamento cirúrgico inicial. A pseudartrose foi considerada se, após 6 meses da osteossíntese, não fossem observados sinais de consolidação clínica e radiográfica da fratura do rádio distal ou do escafóide, 10 conforme avaliação realizada por meio de radiografias em planos ortogonais.

Este projeto recebeu aprovação para realização pelo comitê de ética, com número CAAE: 60074522.2.0000.5505.

Para análise estatística, os dados foram armazenados em uma planilha do Excel® for Mac e, posteriormente, importados para o *Software* SPSS® 23 for Mac. A estatística descritiva dos dados categóricos foi realizada por meio da frequência absoluta de ocorrência e sua respectiva proporção. Os dados contínuos foram analisados quanto à distribuição de forma subjetiva por meio de um histograma e de forma objetiva pelo teste de Shapiro-Wilk. Devido à natureza não simétrica dos dados contínuos, a descrição foi feita utilizando a mediana e os percentis 25 e 75.

As correlações entre as variáveis foram examinadas por meio do teste não paramétrico de correlação de Spearman. Para inferência estatística, valores de p <0,05 foram considerados para o erro do tipo I. A magnitude das correlações foi avaliada usando o Índice de Cohen, 11 classificado da seguinte forma: valores > 0,8 como de grande magnitude, valores entre 0,5 e 0,8 como de média magnitude, e valores entre 0,2 e 0,3 como de pequena magnitude.

## Resultados

Foram analisados 957 prontuários de pacientes submetidos a tratamento cirúrgico no período de janeiro de 2011 a dezembro de 2021, com diagnóstico de fratura na porção distal do rádio. Desses, foram identificados 38 registros com diagnóstico de fratura do rádio distal associada à fratura do escafoide ipsilateral, resultando em uma prevalência de 3,7%. Dois registros foram excluídos por tratarem-se de fraturas do grande arco, restanto 36 pacientes. Após tentativas de contato telefônico e telegrama, 11 pacientes não foram localizados e 2 vieram a óbito por causas não relacionadas. Ao final, 23 pacientes foram avaliados ( Fig. 1 ).

**Fig. 1:** Organograma da obtenção dos pacientes.

No que se refere às características demográficas, observou-se uma distribuição por sexo de 73,9% de homens e 26,1% de mulheres, com 82,6% destros e 7,4% canhotos. Quanto à lateralidade das lesões, contatou-se uma distribuição semelhante, com 56,5% no lado direito e 43,5% no lado esquerdo. A maioria das fraturas do rádio distal foi tratada com placa volar bloqueada (56%), seguida pela placa dorsal bloqueada (21,7%), enquanto outros métodos de fixação foram utilizados para o restante. A fixação das fraturas do escafoide, majoritariamente, foi realizada com parafusos compressivos, e apenas 2 pacientes foram submetidos à fixação com fios de Kirschner ( Tabela 1 ).

**Tabela 1:** Características gerais e tratamento realizado de 23 pacientes com fratura do rádio distal associado a escafoide

	n	%
Sexo		
Feminino	6	26,1
Masculino	17	73,9
Total	23	100,0
Dominância		
Direita	19	82,6
Esquerda	4	17,4
Total	23	100,0
Lateralidade da lesão		
Direita	13	56,5
Esquerda	10	43,5
Total	23	100,0
Método de fixação do radio		
Fixador externo	2	8,7
FK	1	4,34
Hebert	2	8,7
Placa Dorsal	5	21,7
Placa Volar	13	56,5
Total	23	100,0
Método de fixação do escafóide		
FK	2	8,7
Parafuso anterogrado	10	43,5
Parafuso retrogrado	11	47,8
Total	23	100,0

Quanto aos resultados dos questionários, o PRWE apresentou uma média de 35,9 pontos (variação de 14 a 71 pontos), o DASH apresentou uma média de 37,8 pontos (variação de 12 a 78 pontos) e o EVA apresentou uma média de 2,33 durante a atividade (variação de 0,6 a 6,2).

A análise dos aspectos radiográficos revelou uma média de 0,65 mm (−2 mm a 3 mm) para a medida da variância ulnar, 11,43 mm (14 mm a 8 mm) para a altura radial, 20,57 graus (24 graus a 18 graus) para a inclinação radial e 15,30 graus (24 graus a −6 graus) para a inclinação volar. Com base no sistema de classificação AO/ASIF para fraturas da porção distal do rádio, observou-se duas fraturas A3, onze do tipo C2 e dez do tipo C3. Seguindo o sistema de classificação IDEAL, 12 20 pacientes foram considerados potencialmente instáveis e 3 como complexa. Ao avaliar a presença de artrose nos punhos dos participantes por meio da classificação de Knirk-Jupiter, observou-se doze indivíduos como grau 0, nove como grau 1 e apenas dois como grau 2 ( Fig. 2 e Tabela 2 ).

**Fig. 2:** Paciente masculino de 45 anos com história de trauma de alta energia. Tratamento realizado com osteossíntese do rádio distal com placa volar e osteossíntese do escafoide com parafuso compressivo.

**Tabela 2:** Avaliação funcional, classificação e analise de dor de 23 pacientes com fratura de radio distal associado a escafoide

	Média	Desvio padrão	Mediana	1° intervalo interquartil	3° intervalo Interquartil
Tempo de imobilização (semanas)	8,00	2,54	8,00	6,00	8,00
Tempo para o retorno ao trabalho (semanas)	18,81	20,52	12,00	12,00	16,00
Flexão ativa (graus)	50,09	10,37	52,00	42,00	58,00
Flexão passiva (graus)	54,78	9,89	56,00	48,00	63,00
Extensão ativa (graus)	54,70	11,59	54,00	49,00	62,00
Extensão passiva (graus)	59,65	11,87	58,00	53,00	69,00
Desvio radial ativo (graus)	9,96	2,93	10,00	8,00	10,00
Desvio radial passivo (graus)	12,52	2,91	12,00	12,00	14,00
Desvio ulnar ativo (graus)	19,04	5,08	18,00	16,00	20,00
Desvio ulnar passivo (graus)	22,70	5,10	22,00	20,00	24,00
EVA repouso (centímetros)	0,82	1,02	0,30	0,05	1,25
EVA esforço (centímetros)	2,33	1,71	1,40	1,20	3,20
PRWE	35,96	14,03	34,00	27,00	41,50
DASH	37,83	14,77	34,00	30,00	40,00
Variancia ulnar (milímetros)	0,65	1,56	1,00	−1,00	2,00
Altura radial (milímetros)	11,43	1,80	12,00	10,00	12,50
Inclinação radial (graus)	20,57	1,97	20,00	19,50	22,00
Tilt volar (graus)	15,30	6,89	16,00	14,50	19,00
Jamar lado operado (N/Kg)	27,32	7,29	30,34	22,67	32,67
Jamar lado não operado (N/Kg)	31,46	9,92	33,33	24,67	36,67

Dos 23 participantes, apenas 3 apresentaram complicações. Uma infecção de ferida operatória com uma semana de pós-operatório, sendo tratada com antibioticoterapia e evoluindo com resolução completa do quadro. Dois pacientes apresentaram necessidade de retirada da síntese do rádio devido a tenossinovites dos tendões flexores associadas à placa volar bloqueada.

A média de tempo de imobilização foi de 8 semanas (variação de 6 a 12 semanas), enquanto a média de retorno ao trabalho foi de 18,81 semanas (variação de 8 semanas a 1 ano). Um paciente apresentou um intervalo de 2 anos e 3 meses para retornar ao trabalho, em decorrência de um quadro de síndrome dolorosa complexa regional que necessitou de tratamento em conjunto com a equipe especializada em dor ( Tabela 3 ).

**Tabela 3:** Presença de artrose segundo a classificação de Knirk-Jupiter

Classificação de Knirk-Jupter		
G0	9	39,1%
G1	9	39,1%
G2	5	21,7%
G3	0	0,0%

De acordo com o índice de correlação de Spearman, verificou-se um padrão de grande magnitude nos valores medidos de arco de movimento (flexão ativa, extensão ativa, desvio radial ativo, desvio ulnar ativo) ( Tabela 4 ). A correlação entre os questionários PRWE e DASH apresentou um índice de 0,773, considerado de média magnitude, o mesmo ocorrendo entre o PRWE e o EVA durante a atividade do paciente. As demais correlações exibiram efeitos de magnitude pequena ( Tabela 5 ).

**Tabela 4:** Correlações das medidas de arco de movimento

		Flexão passiva	Extensão passiva	Desvio radial passivo	Desvio ulnar passivo
Flexão ativa	Coeficiente de correlação	0,990 *	–	–	–
	Valor de p	<0,01			
Extensão ativa	Coeficiente de correlação	–	0,978 *	–	–
	Valor de p		<0,01		
Desvio radial ativo	Coeficiente de correlação	–	–	0,845 *	–
	Valor de p			<0,01	
Desvio ulnar ativo	Coeficiente de correlação	–	–	–	0,826 *
	Valor de p				<0,01

* Valores acima de 0.8 são considerados valores de grande magnitude.

**Tabela 5:** Correlações dos resultados dos questionários PRWE, DASH e EVA

		PRWE	DASH	EVA repouso	Eva esforço
PRWE	Coeficiente de correlação	–	0,773 *	0,462	0,739 *
	Valor de p		<0,01	0,026	<0,01
DASH	Coeficiente de correlação	–	–	0,210 **	0,418 **
	Valor de p			0,336	0,047
EVA Repouso	Coeficiente de correlação	–	–	–	0,642 *
	Valor de p				<0,01
Eva Esforço	Coeficiente de correlação	0,739 *	0,418 **	0,642 *	–
	Valor de p	<0,01	0,047	<0,01	

* Valores considerados de média magnitude.

** Valores considerados de pequena magnitude.

A correlação se demonstrou de grande magnitude dos valores medidos de arco de movimento (flexão ativa, extensão ativa, desvio radial ativo, desvio ulnar ativo). A correlação dos questionários PRWE e DASH apresentaram um índice de 0,773 considerado de média magnitude, assim como a correlação do PRWE com o EVA em atividade do paciente. As demais correlações se mostraram com magnitudes de efeito pequenos. As correlações estão demonstradas nas Tabela 4 e Tabela 5.

## Discussão

Dentre as fraturas da extremidade distal do rádio, a associação concomitante com fraturas do escafoide ipsilaterais é rara, com uma incidência na literatura de 0,5% a 5%. Todos os pacientes desta série apresentaram mecanismos de trauma de alta energia, sendo a maioria do sexo masculino (73,4%), na quarta década de vida (média de 38,4 anos), em conformidade com o observado na literatura. 2 4 7 9 13 14 15 16 17 18 19 As fraturas apresentaram padrão cominutivo, fortalecendo a hipótese de traumas de alta energia, correspondendo, pela Classificação AO/ASIF, ao subgrupo C (21 pacientes). As fraturas do escafoide, em sua maioria, apresentaram traço simples ao nível da cintura sem desvio, correspondendo a 86%, sendo os 14% restantes fraturas do polo proximal do escafóide.

No presente estudo, ao longo de um período de 10 anos (2011-2021), um total de 957 pacientes com fratura cirúrgica da extremidade distal do rádio foi avaliado, dos quais 36 pacientes apresentaram fratura concomitante e ipsilateral do escafoide, resultando em uma incidência de 3,7%. Dentre esses, 23 pacientes foram incluídos para a analise dos resultados, representando uma amostra significativa dada a baixa prevalência desse tipo de lesão e o cenário da literatura pesquisada, onde foram encontradas apenas cinco publicações com tamanhos amostrais significativos. 2 3 4 13 19 A maior série identificada foi publicada por Vukov et al. (1988), 13 contando com 26 pacientes. No entanto, todos os pacientes foram tratados de modo conservador, o que resultou em uma elevada taxa de complicações (57%).

Neste estudo o tratamento cirúrgico das lesões foi realizado por um único cirurgião, dessa foral a tática cirúrgica para o tratamento das lesões consistiu em seguir sempre a fixação do escafóide por técnica percutânea, anterógrado ou retrógrado para então dar início ao tratamento da fratura do rádio distal através da técnica escolhida para cada caso. Esta estratégia foi adotada uma vez que as fraturas do escafoide, em ampla maioria, eram sem desvio, não necessitando de manobras de redução para a fixação.

Nossos resultados demonstraram que todos os pacientes alcançaram consolidação das fraturas tanto do rádio quanto do escafoide. Somente um paciente enfrentou dificuldades para retornar à atividade laboral original, devido a um quadro de síndrome dolorosa complexa regional. De maneira semelhante, Fowler et al. (2018), 4 em uma série de 23 pacientes tratados cirurgicamente, obtiveram taxas de consolidação do escafoide de 93%. Observou-se que apenas um paciente apresentou fratura desviada do escafoide, e durante a avaliação pós-operatória, somente um caso não apresentou consolidação (correspondendo a um paciente com lesão do plexo braquial ipsilateral).

A taxa de complicações apresentada neste estudo foi de 13%, o que reforça a eficácia do tratamento cirúrgico para os resultados funcionais dos pacientes. Blackburn et al. (2022) 3 realizaram uma revisão sistemática da literatura identificando 20 estudos de séries de casos envolvendo fraturas concomitantes ipsilaterais do escafoide e rádio distal. Essa revisão observou a associação com mecanismos de alta energia e sustentou a necessidade da abordagem cirúrgica para esse tipo de lesão. No entanto, também notou a escassez de trabalhos que apresentassem parâmetros adequados para comparação com avaliação pós-operatória, identificando apenas um estudo que fez uso de questionários como o PRWE.

Dada a baixa prevalência desse tipo de lesão, para fornecer evidências mais robustas, são necessários estudos prospectivos aleatórios multicêntricos. Apesar de nosso estudo ser retrospectivo, houve contribuição para a análise dos resultados ao empregar ferramentas de avaliação funcional validadas e ao demonstrar que tanto o DASH quanto o PWRE foram concordantes na avaliação dos resultados, uma associação até então não identificada na revisão de literatura sobre o tema realizada pelos autores. A utilização desses instrumentos permitiu identificar estatisticamente um índice de correlação de 0,773, indicando que ambos os instrumentos devem ser considerados no escopo de avaliação desses pacientes em estudos futuros.

## Conclusão

Verificamos que as fraturas do rádio distal associadas a fraturas do escafoide foram mais prevalentes em pacientes jovens do sexo masculino e decorreram de traumas de alta energia. O tratamento cirúrgico se mostrou efetivo, conforme avaliação dos desfechos clínicos, radiográficos e funcionais, demonstrando um baixo índice de complicações.
